# Placental microRNA dataset of monochorionic twin pregnancies with and without selective fetal growth restriction

**DOI:** 10.1016/j.dib.2020.105403

**Published:** 2020-03-09

**Authors:** Meng Meng, Yvonne Kwun Yue Cheng, Linda Ling Wu, Piya Chaemsaithong, Maran Bo Wah Leung, Stephen Siu Chung Chim, Daljit Singh Sahota, Wei Li, Liona Chiu Yee Poon, Chi Chiu Wang, Tak Yeung Leung

**Affiliations:** Department of Obstetrics and Gynaecology, Faculty of Medicine, the Chinese University of Hong Kong, Shatin, Hong Kong

**Keywords:** MicroRNA, Microarray, Placenta, Selective fetal growth restriction, Pregnancy, Fetal growth restriction, Twin pregnancy, Mitochondria

## Abstract

To compare the whole genomic microRNA (miRNA) between the selective fetal growth restriction (sFGR) twin and the normally growing (control) co-twin in monochorionic (MC) twin pregnancies. MC twin pregnancies with or without sFGR were recruited, and their placental miRNAs were profiled by microarray. The ratio of the placental miRNA of the sFGR twin to that of the normally larger co-twin were calculated and compared to that of the control twin pairs. The miRNA microarray intensity amongst normal and sFGR large and small twins are shown. The expression data presented here will facilitate other researchers who are working on placental regulation and mechanism in pregnancy complicated by fetal growth restriction.

The dataset supports the research article entitle “Whole genome miRNA profiling revealed miR-199a as potential placental pathogenesis of selective fetal growth restriction in monochorionic twin pregnancies” [1].

Specifications table

**Table:** 

Subject	Obstetrics, Gynecology and Women's Health
Specific subject area	Placental microRNA expression data
Type of data	Table in excel format
How data were acquired	Whole human genome microRNAs microarray.
Data format	Raw data
Parameters for data collection	Density: 8 × 60K
	Coverage: 2549 human miRNAs
Description of data collection	The placental miRNAs were extracted by miRNeasy mini kit and profiled by Agilent Sure Print Whole Genome Human miRNA microarrays. MiRNA features was extracted by Agilent Gene Spring GX software.
Data source location	Institution: Department of Obstetrics and Gynaecology, the Chinese University of Hong Kong
	City/Town/Region: Shatin
	Country: Hong Kong, China
	Latitude and longitude: 22°15′N 114°10′E
Data accessibility	Within the article as a supplementary file
Related research article	Meng Meng, Yvonne Kwun Yue Cheng, Linda Ling Wu, Piya Chaemsaithong, Maran Bo Wah Leung, Stephen Siu Chung Chim, Daljit Singh Sahota, Wei Li, Liona Chiu Yee Poon, Chi Chiu Wang, Tak Yeung Leung
	Whole genome miRNA profiling revealed miR-199a as potential placental pathogenesis of selective fetal growth restriction in monochorionic twin pregnancies
	Placenta
	DOI:10.1016/j.placenta.2020.02.002[1]

## Value of the data

• The data provide an overview of placental differentially expressed miRNAs in monochorionic twin pregnancy complicated by selective fetal growth restriction, a good controlled model to understand the placental mechanism of fetal growth restriction.
• The dataset could be used by researchers working on the investigation of the placenta associated fetal growth restriction in singleton pregnancies and selective fetal growth restriction in twin pregnancies.
• The dataset can be used to further investigate the molecular regulations, functional roles and potential clinical application of the differentially expressed miRNAs in the placental pathogenesis of selective fetal growth restriction.
• Differentially expressed miRNAs identified in this dataset can be used for further downstream analyses. For example (1) Gene Ontology classification for gene function, including molecular function, biological process, and cellular component; (2) KEGG or IPA for miRNA-associated pathway network prediction; and (3) Genes prediction for identification of downstream targets. All these analyses will help to understand the placental pathogenesis of selective fetal growth restriction in monochorionic pregnancy.
• Differentially expressed miRNAs in this dataset can be used to compare with that of other similar published studies, which can explore placental derived miRNAs in monochorionic twin pregnancy complicated by sFGR [2].

## 1 Data description

The dataset contains whole human genome microRNAs data obtained from the microarray profiles of eight pairs of human placentae from monochorionic twin pregnancies complicated with or without sFGR. Five cases are sFGR twin-pairs with case numbers 11, 14, 17, 24, 26, and three cases are normal twin-pairs as control with case numbers 15, 21, 25. Each twin-pair had two fetuses, the smaller and larger fetuses of sFGR twin pregnancies as sFGR-T and sL-T; the smaller and larger fetuses of control twin pregnancy as cS-T and cL-T, respectively. Labels of samples in the dataset were [11sL-T], [11sFGR-T], [14sL-T], [14sFGR-T], [17sL-T], [17sFGR-T], [24sL-T], [24sFGR-T], [26sL-T], [26sFGR-T], [15cL-T], [15cS-T], [21cL-T], [25cL-T], and [25cS-T]. The numbers are referred as case number, sFGR-T as selective FGR twin and sL-T as normally larger co-twin in the monochorionic twin pregnancies; while cS-T and cL-T as the relatively smaller and the relatively larger twins of the control normal twin pairs. MiRNAs intensity of both raw data and normalized data were listed in the dataset from column C to Q, R to AF, respectively. P and Fold Change (FC) value were listed in column AG and AH respectively. The microarray probe sequence and chromosome location of each micRNA probe were also included. Two thousand five hundred and thirty-two miRNAs data were provided in this dataset. Among them, 101 miRNAs were differentially expressed in sFGR when compare to the control group. They were shown in red in the first column.

## 2 Experimental design, materials, and methods

### 2.1 Experimental design

This was a case control prospective study recruiting monochorionic twin pregnancies complicated with sFGR. Monochorionic twin pregnancies with both fetuses having normal estimated fetal weight were also recruited as control group. Monochorionic twin pregnancies complicated with sFGR (sFGR group) were matched with control group by gestational age at delivery.

### 2.2 Subjects

sFGR was suspected when the estimated fetal weight of one of the twins < 10^th^ percentile or z score <-1.282. References of estimated fetal weight and birthweight at different gestational age were based on singletons’ reference for Chinese [3], [4], [5]. After delivery, sFGR was further confirmed when birthweight of one twin was < 10^th^ percentile [3]. The definitions of control group were: (1) monochorionic twin pregnancies, and (2) both fetuses having normal estimated fetal weight and normal birthweight after delivery. In our study, we recruited 17 sFGR and 8 control MC twin pregnancies fulfilled the inclusion criteria. However, we excluded 2 sFGR twin pregnancies with birthweights of both twins below the 10^th^ percentile of the gestation and missed collection of placenta in another 2 sFGR twin pregnancies. Finally, we included 13 sFGR MC twin pregnancies and 8 normal MC twin pregnancies. Amongst all, we randomly selected 5 placentae of the sFGR twin-pairs and 3 placentae of the normal twin-pairs for miRNA microarray profiling, and we used all placentae for validation. Here we only report miRNA data, containing 5 cases of sFGR twin-pairs (cases 11, 14, 17, 24, 26), and 3 cases of normal twin-pairs as control (cases 15, 21, 25) in the supplementary table. The other sFGR and control cases were only used for validation. They did not have microarray data.

### 2.3 Placental tissue collection and preparation

Like the study of Wen et al. [2], the monochorionicity of each placenta was confirmed immediately after delivery. The territories of smaller twin and larger twin were recognized by their amniotic membranous septum and vascular distribution. For smaller twin, at least 3 pieces of placentae were collected within it is territory. And then did the same to the larger twin. All fresh placentae were collected for each recruited case. After that, chorions were divided from the decidua and amnion of placentae [6]. Only chorions were used for the experiments. Samples were collected within 30 min after delivery. For miRNA microarray profiling, the placental chorions were immersed in RNAlater (Ambion, Austin, TX, USA) immediately. The functions of RNAlater are to prevent RNA degradation. Tissues were kept at 4 °C overnight. Then RNAlater was removed and the placental chorions were aliquoted and stored at −80 °C until RNA isolation.

### 2.4 miRNA microarray

Placental total RNAs (containing total miRNAs) of the eight recruited twin-pairs were extracted from 50 mg of homogenized placental tissue by miRNeasy mini Kit (Qiagen, Valencia, CA, USA). The quality of RNAs was checked by Agilent 2100 Bioanalyzer (Agilent technologies Inc., Palo Alto, CA, USA) and NanoDrop ND-2000 spectrophotometer (Thermo Fisher Scientific, Waltham, MA, USA). Only the RNAs with high purity and integrity were used for microarray assay. Agilent SurePrint Human miRNA microarray chips were used to profile the whole human miRNAs. This 8 × 60 K microarrays covered 2549 human miRNAs. Microarray data analysis was carried out by Agilent GeneSpring GX software. The quality of raw data was checked first, data which didn't pass the quality control were excluded. The microarray data of one placenta sample from the smaller twin in control group ([21cS-T]) didn't pass the quality control. In order to avoid the analysis bias, the data of [21cS-T] was excluded and removed from the raw data and analysis. All other passed the quality check, raw miRNA expressions were transformed into normalized data by quantile normalization using median. MiRNAs exhibited FC >1.5 and *P*-value <0.05 were identified as differentially expressed miRNAs.

## Appendix Supplementary materials

Image, application 1
